# Endothelial Keratoplasty: From DLEK to DMEK

**DOI:** 10.4103/0974-9233.61210

**Published:** 2010

**Authors:** Mark M. Fernandez, Natalie A. Afshari

**Affiliations:** 1 Department of Ophthalmology, Cornea and Refractive Surgery, Duke University Eye Center, Durham, NC, USA

**Keywords:** Descemet Stripping and Automated Endothelial Keratoplasty, DSEK, Deep Lamellar Endothelial Keratoplasty, Descemet's Membrane Endothelial Keratoplasty, Endothelial Keratoplasty

## Abstract

The last decade has heralded a revolutionary shift in the treatment of corneal endothelial disease. Only 15 years ago, the only surgical treatment for pseudophakic bullous keratopathy and Fuchs dystrophy was penetrating keratoplasty (PK). Although used successfully for over a century, PK requires many months of refractive adjustments before the eye achieves visual stability. Starting with the advent of posterior lamellar keratoplasty in the late 1990s, a number of procedures have been developed, refined, and widely adopted, which have given patients faster recoveries and improved globe stability in comparison to traditional corneal transplantation. Each iteration of endothelial keratoplasty (EK) has involved the increasingly selective transplantation of corneal endothelial cells. Preliminary results of the most recent form of EK, Descemet's membrane EK, suggest that pure endothelial cell transplantation is on the horizon.

## INTRODUCTION

Two of the most frequent indications for corneal transplantation in the United States are Fuchs dystrophy and pseudophakic Bullous Keratopathy (PBK).^1^ These diseases are derangements of the corneal endothelium that have been treated with penetrating keratoplasty (PK) for decades. Recent advances in endothelial keratoplasty (EK), the selective transplantation of components of the cornea instead of full-thickness keratoplasty, have revolutionized the treatment of these diseases, with improved recovery times and visual outcomes.

Although it has been used successfully for over a century, PK has many shortcomings. A period of several months after surgery is usually required to attain visual stability as astigmatic error changes every time a suture is removed.^2^ Meanwhile, the presence of an avascular graft-to-wound interface lowers globe stability and maintains the risk of dehiscence years after surgery.^3^

In EK, only a portion of the recipient's posterior cornea is removed. Few or no sutures are used and minimal astigmatism is induced. A smaller graft is transplanted, meaning that less foreign antigen is introduced to the donor. Meanwhile, the absence of sutures at the interface between graft and host tissues lowers the incidence of vascular ingrowth and graft rejection. The absence of a large full-thickness penetrating wound also lowers the risk of dehiscence. New techniques in EK enable the surgical treatment of corneal endothelial disease with a lower risk of rejection, improved globe stability, and faster visual recovery than traditional full-thickness corneal transplantation.

## ENDOTHELIAL DISEASE

The adult human cornea averages 540 µm in thickness,^4^ with the following layers from anterior to posterior: Epithelium, Bowman's membrane, stroma, Descemet's membrane, and endothelium. The cornea remains in a state of deturgescence, maintained by endothelial cell Na^+^/K^+^ ATPase and by tight junctions between endothelial cells that limit ingress of fluid into the stroma. By maintaining an optimum level of corneal hydration, endothelial cells preserve the ordered arrangement of collagen, which is crucial for corneal transparency.^5^ When endothelial cell density is low, the loss of tight junctions between cells allows more fluid to enter the stroma. The endothelial cells that remain may have a higher concentration of Na^+^/K^+^ ATPase in an effort to compensate for the loss.^4^

The average human cornea has an endothelial cell density of 5,000-6,000 cells/mm^2^ at birth, decreasing to 2,500-3,000 cells/ mm^2^ by adulthood. There is an average cell loss of 0.6% per year.^4^ Corneal edema appears at 700-400 cells/ mm^2^.^4^,^6^ Adult human corneal endothelial cells are arrested in the G^1^ phase of the cell cycle and do not undergo mitosis.^7^ Therefore, lost cells cannot be physiologically replaced.

In Fuchs dystrophy, the total number of endothelial cells is low and existing cells may not function properly. Descemet's membrane thickens and develops excresences known histopathologically as guttae. Stromal edema develops and corneal thickness may increase to over 1,000 µm. When edema is severe, the corneal epithelium can detach from its basement membrane, creating painful bullae on the anterior surface of the cornea.^5^,^8^

PBK is endothelial cell loss caused by surgery in the anterior chamber. If the corneal endothelium is damaged during surgery (as often occurs during cataract extraction),^6^ the same spectrum of symptoms as found in Fuchs dystrophy can develop.

## EVOLUTION OF ENDOTHELIAL KERATOPLASTY

### Early efforts

In the 1960s, Dr. Jose Barraquer described a method of EK using an anterior approach via a LASIK flap.^9^ After a partial thickness flap was cut with a microkeratome, the posterior cornea consisting of stroma, Descemet's membrane, and endothelium was trephined and replaced with a donor graft that was sutured in place. The flap was then replaced and also sutured. This lowered the amount of donor tissue grafted and eliminated the need for full-thickness incisions, but it required multiple sutures. Irregular postoperative astigmatism and vascular ingrowth remained a limitation of this procedure.

### Foundations of modern endothelial keratoplasty

Gerrit Melles, MD, laid the foundation of modern EK in 1998.^10^ In a procedure he called posterior lamellar keratoplasty (PLK), Melles dissected the posterior lamella, Descemet's membrane, and endothelium through a 9-mm sclerocorneal incision. A donor button consisting of posterior stroma, Descemet's membrane, and endothelium was then inserted and held in place by an air bubble while the patient lay supine.^11^ PLK was subsequently adopted by Mark Terry, MD, in the United States, who termed the procedure deep lamellar endothelial keratoplasty (DLEK).

Melles revised the procedure by using a 5-mm incision and bending the donor tissue to enable insertion.^12^ Terry and Ousley reported similar results between patients treated with DLEK using this 5-mm incision and the older 9-mm incision in a large prospective study.^13^ The PLK/DLEK procedure showed promise because visual acuity recovered rapidly and eyes had only small increases in astigmatism 6 months after surgery. In a prospective series involving 100 eyes, Terry and Ousley reported an average 6-month postoperative astigmatic error of 1.34 ± 0.86 D. This represented an average increase of 0.28±1.08 D from pre-operative levels.^14^ This group of patients had a best spectacle-corrected visual acuity (BSCVA) of 20/46 6 months after surgery, an improvement from the pre-operative average BSCVA of 20/104. Donor endothelial cell density at 6 months was 2,140±427 cells/mm^2^, representing an average cell loss of 25% from pre-operative counts.

In a separate study of 20 eyes with Fuchs dystrophy treated with DLEK, Ousley and Terry reported no significant change in spherical refraction or astigmatic error between 1 and 2 years after surgery.^15^ The same study reported a 2-year endothelial cell density of 2,151±457, slightly lower than the 1-year density of 2,335±468. Melles reported that 15 consecutive eyes treated with PLK had 1,047±425 cells/mm^2^ 3 years after surgery, down from 2,126±548 at 6 months.^16^ These promising results were tempered by the technical difficulty of the procedure, however, which necessitated the manual dissection of both donor and host stromal beds.

### Descemet's transplantation conceived

In 2004, Melles *et al*. described a method to dissect only the Descemet membrane from the recipient eye, leaving the posterior lamella intact.^17^ This followed a proof-of-concept study, wherein his group transplanted a Descemet's membrane via a 5-mm, self-sealing incision in 30 cadaver eyes.^18^ The average postoperative astigmatic error was 1.0±0.6 D, only slightly higher than the pre-operative astigmatic error of 0.7±0.3 D. This procedure did not require manual dissection of the recipient stromal bed, allowing excellent apposition of donor and recipient tissue with minimal, if any, interface haze. The drawback of this procedure was that a Descemet's membrane without stromal support lacks rigidity and positioning the graft within the anterior chamber was technically difficult.^19^ The lack of stromal support also resulted in spontaneous rolling of the membrane, which was thought to increase damage to the endothelium. The estimated endothelial cell damage immediately after surgery was approximately 2-4%.

### The birth of Descemet's stripping automated endothelial keratoplasty

An improvement to DLEK that developed after Melles demonstrated his technique to selectively remove Descemet's membrane from its stromal bed was DSEK, or Descemet's Stripping EK. In this procedure, pioneered by Mark Gorovoy, MD, and Francis Price Jr., MD,^20^ host Descemet's membrane is stripped using the Melles technique.^17^ A donor posterior cornea is then cut with a microkeratome to leave a donor button consisting of posterior stroma, Descemet's membrane, and endothelium. This button is folded once, inserted into the anterior chamber via a small (∼5 mm) incision, and opened by gentle manipulation with a cannula. As it is in DLEK, the graft is held in place by an air bubble until suction created by Na^+^/K^+^ ATPase molecules in the donor endothelium adheres the graft to the host.

In a prospective study of 50 eyes, Price *et al*. reported that 76% of the eyes treated with DSEK corrected to 20/50 or better, and 62% corrected to 20/40 or better. The mean postoperative manifest cylinder was unchanged from the mean pre-operative manifest cylinder.^21^ Many eyes treated with DSEK did not correct to 20/20, however, and this was attributed to haze at the graft-host interface.

To improve the graft-host interface, the donor cornea may be dissected with an automated microkeratome in a procedure termed DSAEK, or Descemet's Stripping Automated Endothelial Keratoplasty. DSAEK has been shown to have faster visual recovery, lower postoperative astigmatism, and a lower incidence of graft failure than PK.^22^ In a comparison of PK with DSAEK at over 1 year after transplantation, DSAEK had a statistically insignificant higher rate of repeat grafting at 15 months and a statistically higher rate of endothelial loss at 12 months (38% *vs*. 20%).^23^

Regardless of this, DSAEK has been highly successful and widely adopted. In a cohort study of 12 patients who underwent DSAEK surgery in one eye and PK in the other, all patients reported higher satisfaction with the DSAEK procedure and achieved better uncorrected and best-corrected visual acuities.^24^

### A return to selective Descemet's transplantation

Following the widespread adoption of DSAEK surgery, the Melles group revisited selective Descemet's membrane transplantation and reported the results of a new procedure, Descemet's Membrane Endothelial Keratoplasty (DMEK).^25^ In DMEK, the donor Descemet's membrane was stripped from a corneoscleral rim and injected into the host anterior segment, which had been stripped of its own Descemet's membrane, via a 3-mm clear corneal incision. The membrane was unrolled using pneumatic and fluidic manipulations and apposed to the recipient posterior stroma using the same air bubble technique as pioneered in the prior techniques.

The initial results were encouraging; of 10 eyes transplanted, four had best-corrected visual acuity of better than 20/40 1 week after surgery and six saw greater than 20/40 at postoperative week 6. Moreover, this simplified technique negated the need for an automated microkeratome to smooth a stromal graft, meaning that this technique would be accessible to a greater number of surgeons. The Melles group subsequently presented their first 50 cases of DMEK; of those eyes where the Descemet's graft adhered (*n = *40, 80%), 75% achieved BSCVA of 20/25 or better within 3 months.^26^

In the December of 2009, 2 months after the Melles paper, Price *et al*. reported their prospective study of 60 DMEK procedures in 56 eyes in the United States. Their results were similar to the Melles study; the Price group reported 63% eyes with a visual acuity of 20/25 or better and 94% with vision of 20/40 or better at 3 months.^27^ This was significantly better than the same groups results with DSAEK surgery.

Initial endothelial cell counts have been comparable to PK and DSAEK. The Melles group reported an average endothelial cell density of 1,850 cells/mm^2^ at 6 months after surgery and 1,680 cells/mm^2^ at 12 months.^28^ The Price group reported a mean endothelial cell loss of 30% at 3 months.

DMEK presents many significant improvements over DSEK. First, the number of eyes achieving 20/25 or better vision with DMEK is much higher than typically expected in DSEK, where 20/25 visual acuity is rare but patients are typically very pleased with the results. Second, this procedure eliminates the need for an automated microkeratome, increasing its ease of adoption, especially in the developing world. Finally, this graft does not include corneal stroma, suggesting that a myopic shift would not occur as it does in DSEK.

## CONCLUSION

**Figure 1:**
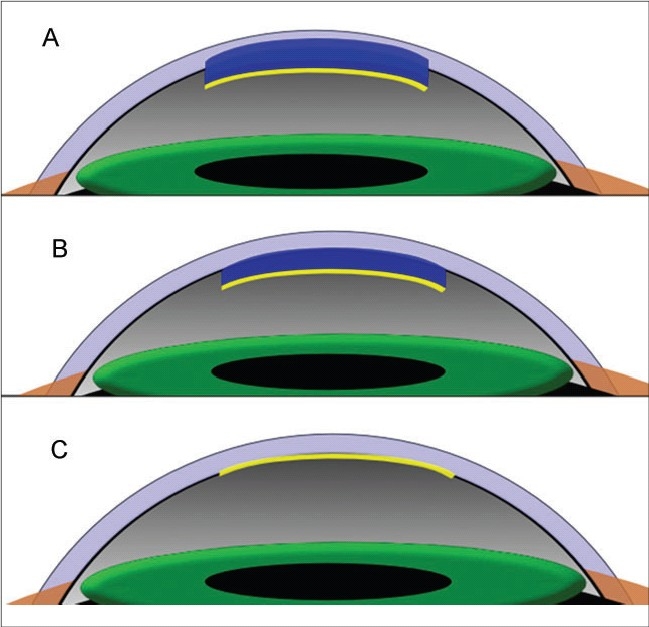
(A) In deep lamellar endothelial keratoplasty, Descemet's membrane and posterior corneal stroma is removed. It is replaced by a graft consisting of posterior stroma and Descemet's membrane; (B) In Descemet's stripping automated endothelial keratoplasty, only the host Descemet's membrane is removed. This is replaced by a donor graft of posterior stroma and Descemet's membrane; (C) In Descemet's membrane endothelial keratoplasty, only the host Descemet's membrane is removed and replaced with the donor Descemet's membrane. Corneal stroma is not transplanted.

The past decade has heralded a revolution in the surgical treatment of diseases of the corneal endothelium. For over 100 years, patients with Fuchs dystrophy and bullous keratopathy were offered only PK, which entailed months of follow-up with multiple changes in refractive error. The rapid and widespread adoption of DLEK and DSAEK in the past few years has provided hundreds of patients with a minimally invasive surgical treatment producing excellent uncorrected vision and globe stability. The newest iteration of EK is DMEK, a minimally invasive treatment with the promise of minimal induced refractive error and excellent uncorrected vision within only a few weeks of surgery [Figure 1].

Each iteration of EK has brought corneal surgeons one step closer to pure endothelial cell transplantation or even more innovative treatments for diseases of the endothelium. The Melles group, for example, has recently reported spontaneous corneal clearing after DMEK with a graft that dislocated permanently-could corneal clarity be achieved in the absence of a permanent graft?^29^ The future is bright for endothelial transplantation, which promises to be the standard of care for PBK and Fuchs dystrophy in the century to come.
